# Shift in Sex Ratio: Male Numbers Sink in Great Lakes Community

**Published:** 2005-10

**Authors:** Ernie Hood

Sex ratio—the proportion of male to female live births—can be an important indicator of the reproductive health of a population, whether animal or human. This figure is typically fairly constant. For example, the worldwide human sex ratio ranges from 102 to 108 male births for every 100 female births; in other words, male babies make up about 50.4–51.9% of live births worldwide. Now, however, investigators have documented a significant skewing of the human sex ratio in a population located in a heavily polluted Great Lakes area **[*EHP* 113:1295–1298]**.

In response to concerns about a shifting sex ratio among members of the Aamjiwnaang First Nation community near Sarnia, Ontario, a team of Canadian researchers examined birth records for the group from the years 1984–2003 as part of a broader community-based investigation. The researchers discovered that, as community members had suspected, there had been a significant and precipitous shift in the sex ratio.

The expected sex ratio in Canada is 51.2% male babies to 48.8% female babies. For the period 1984–1992, that ratio held fairly constant among this community. In the period 1993–2003, however, male babies made up only 41.2% of live births. The five-year period from 1999 to 2003 showed an even sharper decline, with male babies making up 34.8% of live births. According to the researchers, although there is normal variation in sex ratio within populations, the deviation in this case appears to be outside the normal range.

Although there is as yet no direct evidence linking this human sex ratio decline to environmental exposures, the circumstantial evidence suggests there may be a connection. The Chippewas of the Aamjiwnaang reserve reside within the St. Clair River Area of Concern, situated immediately adjacent to several large petrochemical, polymer, and chemical industrial plants. The area is one of Canada’s largest concentrations of industry. Prior soil and sediment assessment has shown that the reserve land is heavily contaminated with pollutants such as polychlorinated biphenyls, polyaromatic hydrocarbons, hexachlorobenzene, mirex, a variety of potentially toxic metals, volatile organic compounds, phthalates, and dioxins; many of these are known or suspected endocrine disruptors.

As the investigators point out, past studies have documented reproductive outcomes in wildlife populations within the same region, including reduced hatching success, altered sexual development, and changes in sex ratios. Scientific suspicion has long been focused on environmental endocrine disruptor exposures as the root cause of these effects.

The authors acknowledge that there are many other potential factors that could influence the declining sex ratio they describe. But the combination of close proximity to industrial facilities emitting known endocrine-disrupting chemicals and the documented adverse reproductive outcomes in wildlife populations in the region leads them to conclude that further investigations are warranted into the types and routes of chemical exposures—via air, water, food, soil, and sediment—for this population. A community health survey designed to explore health concerns among residents of the reserve is in progress, including information on potential covariates that may influence the sex ratio, such as parental age or smoking.

## Figures and Tables

**Figure:**
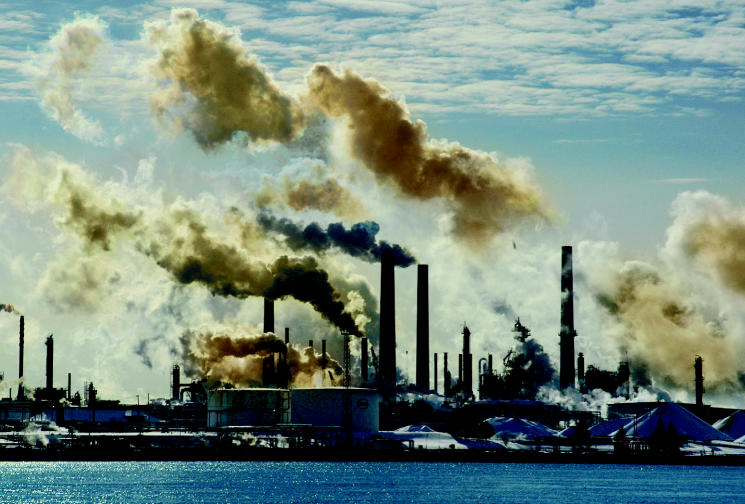
Chemical culprit? The Sarnia–Lambton area in Ontario is home to Chemical Valley as well as the Aamjiwnaang First Nation community, which has experienced a significant skewing of the ratio of male to female babies born in recent years, leading some to question whether environmental exposures may be to blame.

